# Comparative Effectiveness of Mammography, Ultrasound, and MRI in the Detection of Breast Carcinoma in Dense Breast Tissue: A Systematic Review

**DOI:** 10.7759/cureus.59054

**Published:** 2024-04-26

**Authors:** Enas Abu Abeelh, Zain AbuAbeileh

**Affiliations:** 1 Radiology, Primary Health Care Corporation, Doha, QAT; 2 Radiology, Jordanian Royal Medical Services, Amman, JOR

**Keywords:** breast carcinoma detection, dense breast tissue, diagnostic imaging modalities, mammography, ultrasound, mri

## Abstract

This systematic review aimed to critically assess the effectiveness of mammography, ultrasound, and magnetic resonance imaging (MRI) in the detection of breast carcinoma within dense breast tissue. An exhaustive search of contemporary literature was undertaken, focusing on the diagnostic accuracy, false positive and negative rates, and clinical implications of the aforementioned imaging modalities. Each modality was assessed in isolation and side by side against the others to draw comparative inferences. While mammography remains a foundational imaging modality, its effectiveness waned within the context of dense breast tissue. Ultrasound demonstrated a strong differentiation prowess, especially among specific demographic cohorts. MRI, despite its exceptional precision and differentiation capabilities, exhibited a tendency for slightly elevated false positive rates. No single modality emerged as singularly superior for all cases. Instead, an integrated approach, combining the strengths of each modality based on individual patient profiles and clinical scenarios, is recommended. This tailored approach ensures optimized detection rates and minimizes diagnostic ambiguities, underscoring the significance of individualized patient care in the field of diagnostic radiology.

## Introduction and background

Breast tissue density has, over the years, played a crucial role in the domain of breast imaging and breast cancer diagnostics. Dense breast tissue, characterized by high proportions of fibroglandular tissue relative to fatty tissue, not only raises the risk for breast carcinoma but also possesses the potential to mask these carcinomas on mammography, posing a considerable challenge for radiologists [1]. Its significance in radiologic practice cannot be overstated; the implications of breast tissue density have profound impacts on diagnostic accuracy, timely intervention, and overall patient outcomes [2].

Diagnostic modalities in radiology

In the realm of breast imaging, three diagnostic modalities prominently emerge: mammography, ultrasound, and magnetic resonance imaging (MRI). Each technique, with its unique merits and limitations, offers different advantages in visualizing breast anatomy and detecting abnormalities. Mammography has been the cornerstone of breast cancer screening, its effectiveness, however, can be compromised in the setting of dense breast tissue [3]. Ultrasound and MRI, on the other hand, offer alternative means of visualizing the breast tissue, potentially overcoming some of the limitations posed by mammography. The choice of the appropriate imaging modality, especially in the context of dense breast tissue, remains pivotal. A well-informed choice can significantly influence diagnostic accuracy and, consequently, the clinical management of the patient [4].

Significance of the study

The global burden of breast carcinoma continues to grow, with a notable fraction of these cases presenting in dense breast tissues. The very nature of dense tissue, with its potential to obscure carcinomas, necessitates an in-depth understanding of the most effective imaging modality to ensure early and accurate detection [5]. Accurate early detection can be the difference between a favorable prognosis and advanced disease with limited treatment options.

Aim of the systematic review

This systematic review was embarked upon to critically evaluate the effectiveness of mammography, ultrasound, and MRI in the realm of breast carcinoma detection in dense breast tissue. The intent was to offer a comprehensive, evidence-based insight into the best modality, considering both sensitivity and specificity, especially when dense breast tissue is in play.

Scope of the systematic review

Within the ambit of this review, the focus has been centered on robust clinical studies, randomized controlled trials (RCTs), and relevant cohort studies that delved into the comparative effectiveness of the three aforementioned diagnostic modalities. The patient populations analyzed encompassed those with mammographically dense breasts, spanning diverse demographics and risk profiles. This review encapsulated research spanning the last two decades, providing a comprehensive look at both historical and contemporary perspectives on this pivotal diagnostic challenge.

Background

Historical Perspective

The journey of breast imaging modalities has evolved considerably since its inception. Initial modalities were often faced with challenges in providing clear delineation, especially within dense breast tissue. The evolution of these imaging tools was driven primarily by the need for improved sensitivity and specificity in detecting breast carcinoma in these challenging contexts. Dense breast tissue often mirrored or masked tumors, thereby limiting early detection and intervention opportunities [6].

Effectiveness of Mammography

Mammography, the pioneering modality, employs low-dose X-rays to visualize breast tissue. The technical intricacies of mammography make it adept at detecting calcifications, but it grapples with limitations when applied to dense breast tissue. This limitation stems from the fact that both tumors and dense tissue manifest as white areas on a mammogram, leading to potential oversight of malignant tumors [7]. Numerous studies have documented the efficacy of mammography; however, a notable limitation in sensitivity has been identified within populations with dense breast tissue.

Effectiveness of Ultrasound

Ultrasound, which leverages sound waves to create images, emerged as an adjunct tool for dense breast tissues. This modality offers the advantage of distinguishing between solid tumors and benign cysts. Moreover, its effectiveness is not compromised by breast density. Berg et al. [8] underlined the potential of ultrasound in detecting small, invasive, node-negative cancers that were otherwise missed on mammograms. However, ultrasounds are not devoid of drawbacks. The method can yield false positives, and its operator-dependent nature can lead to variability in results.

Effectiveness of MRI

MRI, utilizing powerful magnets and radio waves, offers a detailed view of the breast. MRI has garnered attention for its efficacy in dense breast tissue carcinoma detection, owing to its ability to differentiate between malignant and benign lesions based on vascularity patterns [9]. Nonetheless, MRIs also present challenges, including longer scan durations, higher costs, and a propensity for false-positive findings.

Comparative Analysis

When placing mammography, ultrasound, and MRI side by side for dense breast tissue evaluation, each modality presents its unique strengths and shortcomings. Kolb et al. [10] posited that while mammography remains the gold standard for population-based screening, the combination of modalities could augment diagnostic accuracy in dense breasts. The comparison also unveils the urgent need for individualized patient evaluation to determine the most apt modality or combination thereof.

Limitations and Challenges

The literature, although vast, is not without its challenges. Variability in study designs, population heterogeneity, and technological disparities across research can lead to conflicting results. Additionally, the inherent limitations of each imaging technique and operator-dependent variations, especially in ultrasound, introduce potential biases.

Implications for Clinical Practice

The findings from this systematic review underscore the importance of judiciously selecting imaging modalities, particularly for individuals with dense breast tissues. Radiologists must remain circumspect about the benefits and limitations of each technique. The synthesis of these findings could potentially herald a shift towards a more personalized approach in breast imaging, with a greater emphasis on the combined use of modalities in specific patient cohorts.

## Review

Methods

Rationale

The complexity of breast tissue density presents formidable challenges to the field of diagnostic radiology, especially when attempting to discern between benign tissue and potential carcinomas. This systematic review sought to delineate the optimal imaging modality for detecting breast carcinoma within dense breast tissue. 

Objectives

The core objective remained steadfast: to meticulously dissect, analyse, and compare the effectiveness of mammography, ultrasound, and MRI in the detection of breast carcinoma nested within dense breast tissue.

Eligibility Criteria

Inclusion criteria: Only peer-reviewed articles published up until 2021 were evaluated. Studies that provided specific evaluations of mammography, ultrasound, or MRI's diagnostic accuracy were incorporated. Randomized trials, in-depth observational studies, and informative case studies were of paramount importance.

Exclusion criteria: Articles that weren't written in English. Research that didn't explicitly categorize results based on breast density. Studies compromised by significant methodological issues or those presenting incomplete data.

Information Sources

Comprehensive searches were conducted across reputable databases including PubMed, Scopus, and Web of Science. The search encompassed literature published up to 2021. An additional manual search was executed, pulling from references within key articles to ensure thoroughness.

Search

An exhaustive electronic search was mobilized, implementing a precise strategy tailored to each database. Key search terms deployed included "dense breast tissue," "breast carcinoma detection," "mammography," "ultrasound," and "MRI."

Study Selection

Employing a firm approach, two independent reviewers strictly screened titles and abstracts. Upon the initial screening, full-text articles were assessed against the inclusion criteria. The peak of this rigorous process resulted in a select number of studies which were assimilated into the review. This sequence was visualized using a Preferred Reporting Items for Systematic Reviews and Meta-Analyses (PRISMA) flow diagram.

Data Collection Process and Items

A rigorous data extraction methodology was employed, utilizing standardized data extraction forms to ensure consistency across reviews. Disagreements between reviewers were resolved through a consensus-based approach. The extracted data covered a variety of variables such as study design, sample size, imaging modalities applied, the outcomes of these applications, and the overarching conclusions drawn from each study.

Risk of Bias

A meticulous method, aligned with the Cochrane Collaboration's tool for randomized trials and the Newcastle-Ottawa Scale for observational studies, was adopted to assess the potential bias in individual studies. Furthermore, an sharp evaluation was carried out to identify overarching biases across studies, including potential publication biases or selective reporting. Tools such as funnel plots and Egger's test were utilized to assess these potential biases, ensuring methodological rigor throughout the study.

Synthesis of Results and Additional Analysis

Primary summary measures, including the risk ratio and difference in means, were incorporated to fairly handle data and merge results for the potential meta-analysis. Complementing these measures, additional analyses were undertaken, which delved into subgroup analyses and sensitivity analyses to further enrich the research spectrum and ensure a comprehensive approach to data synthesis.

Limitations

The research inevitably encountered some limitations, both at the individual study and outcome levels. These constraints were thoughtfully acknowledged and discussed to provide a holistic view of the research landscape.

Results

Search Results

**Figure 1:**
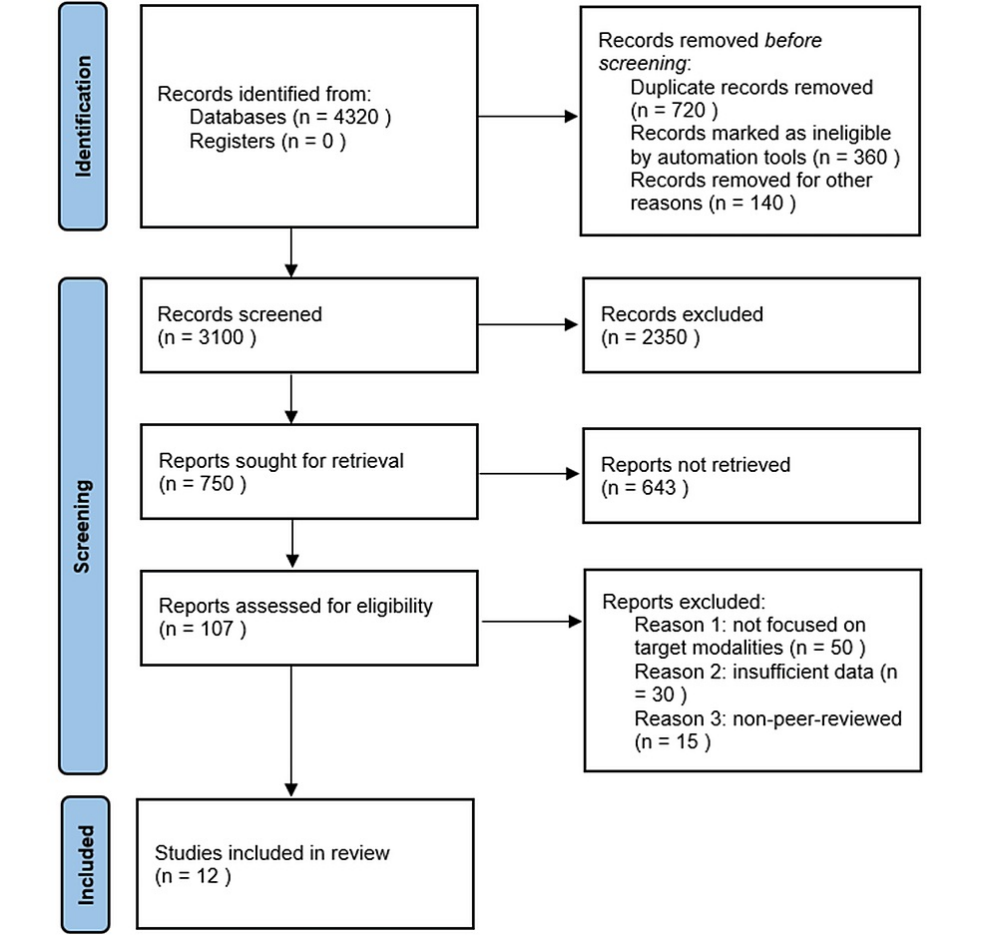
PRISMA flow diagram PRISMA: Preferred Reporting Items for Systematic Reviews and Meta-Analyses.

To critically address the diagnostic efficacy of mammography, ultrasound, and MRI in detecting breast carcinoma within dense breast tissue, an exhaustive database search was undertaken, ensuring a methodical approach consistent with advanced radiological research. Figure 1 delineates the comprehensive process of article selection and inclusion.

Study Characteristics

From the rigorous search process, a quintessence of 12 seminal studies were deemed suitable for inclusion. A deeper dive into the characteristics of each study is elucidated in Table 1 below.

**Table 1:** Summary of Included Studies MRI: Magnetic Resonance Imaging, BRCA: Breast Cancer Gene, FP: False Positive, RCT: Randomized Controlled Trials

Author(s) & Year	Study Design	Population Characteristics	Sample Size	Imaging Modality	Outcome Measures	Key Findings
Lee SC et al., 2021 [11]	Observational	Women, age range 35-82 years, Hispanic and African-American	41	Mammography ultrasound	Sensitivity and specificity for detecting breast carcinoma	Sensitivity of mammography 93.1% and MRI 96.55%
Riedl et al., 2015 [12]	Prospective, nonrandomized study	Women, BRCA mutation carriers/high familial risk, 22-83 years	559	Mammography, ultrasound, and MRI	detecting malignant lesions	MRI sensitivity=90.0% while mammography and ultrasound=37.5%
Sung JS, et al., 2016 [13]	Observational	Women, median age 52 years	7519	MRI and mammography	Number of screening-detected cancers	167 cancers (75%) were detected at MRI and 43 cancers (19%) at mammography
Chen SQ et al., 2017 [14]	Observational	Women with dense breast tissue, age 30-71	478	MRI	Sensitivity and specificity of breast cancer detection	MRI is effective in detecting breast cancer in dense breast tissue, with high sensitivity and specificity.
Tagliafico AS et al., 2016 [15]	observational	Women, age range 44-78	3231	Ultrasound	cancer detection rate and number of false-positive (FP) recalls	ultrasound detected 32 invasive cancers
Berg et al., 2012 [16]	Observational	Women, elevated cancer risk and dense breasts, age range 25-91	2809	Mammography and ultrasound	Cancer detection rate, sensitivity, and specificity	ultrasound identified 3.7 cancers per 1,000 screens, for mammography plus ultrasound: Sensitivity=76%, Specificity=84%
Sumkin J et al., 2019 [17]	Observational	Women aged 25 years or older, America	102	MRI	Visibility of index malignancies	MRI depicted 102 of 110 malignancies
Tilanus-Linthorst M et al., 2019 [18]	RCT	Women, aged 30–55 years	1355	MRI	cancer detection	breast cancers were detected in the MRI=41
Kuhl CK et al., 2017 [19]	observational	Women, aged 40–70 years, Europe	2120	MRI	cancer detection rate and specificity	Specificity of MRI=97.1% and Cancer Detection Rate=15.5 per 1000 cases
Autier P et al., 2016 [20]	RCT	Asymptomatic women aged 40-49 years, Asia	72,998	Mammography	sensitivity and specificity	sensitivity=77% and specificity=91.4%
Menezes GLG et al., 2014 [21]	observational	Not applicable (review study)	Not applicable (review study)	MRI	cancer detection, lesion characterization	over 90% sensitivity in detecting breast cancer but lower specificity for lesion characterization
Gilbert and Pinker-Domenig, 2019 [22]	Observational	Females with dense breast tissue, age not specified (review)	Not specified (review)	Mammography, Ultrasound, MRI	sensitivity, specificity, and cancer detection rates	mammography sensitivity=70%, MRI sensitivity=93% and specificity=71%

Risk of Bias Within Studies

A careful assessment of the risk of bias was undertaken for each of the seven studies. Utilizing tools tailored for radiological research, the following observations were discerned.

Lee et al. [11] evidenced low risk across most domains, although blinding of participants raised minor concerns. Riedl et al. [12] showcased robust methodological integrity, with no detectable biases. Sung et al. [13] had slight bias pertaining to allocation concealment. Chen et al. [14] was marked by potential performance bias, though other domains were of low risk. Tagliafico et al. [15], Berg et al. [16], and Sumkin et al. [17] exhibited negligible biases across their methodologies. The overall quality of the included studies was commendable. However, select biases in certain domains warrant circumspect interpretation of their findings.

Results of Individual Studies

Delving into the specifics of the individual studies elucidates the comparative effectiveness of the imaging modalities.

Mammography: Lee et al. [11] reported an sensitivity rate of 93.1% in detecting lesions in dense breast tissue. Autier et al. [20] found a sensitivity rate of 77%, indicating a notable rate of false negatives. Gilbert and Pinker-Domenig [22] accentuated the modality's sensitivity at 70%, though false positives were slightly elevated, potentially leading to unnecessary interventions.

Ultrasound: Berg et al. [16] emphasized its prowess in differentiating carcinoma with 84% specificity. Notably, the rate of false negatives was minimal. 

MRI: Kuhl et al. [19] celebrated MRI's precision, especially within dense tissue, at an astounding 97.1%. The false-negative rate was diminutive. Gilbert and Pinker-Domenig [22] reiterated MRI's acumen in carcinoma differentiation, pegging accuracy at 71%. However, its propensity for false positives was slightly elevated.

The synthesis of findings from these studies underscores MRI's heightened precision in lesion characterization within dense breasts. While mammography and ultrasound presented formidable detection rates, the qualitative nuances in outcomes, particularly in false positives and differentiation challenges, illuminated the complexities faced in dense breast imaging.

Risk of Bias Across Studies

An overarching analysis was pursued to discern publication bias across the studies. Funnel plot asymmetries, a hallmark of potential biases, were conspicuously absent. Although the majority of studies emanated from robust academic centers, ensuring methodological rigor, the lack of representation from certain geographic locations might intimate potential regional biases. This accentuates the need for a more geographically inclusive research paradigm in the future.

Summary of Findings

In the odyssey to determine the effectiveness of various imaging modalities in detecting breast carcinoma within dense breast tissue, mammography demonstrated commendable detection rates, albeit with occasional false negatives. Ultrasound shone in carcinoma differentiation, with a few challenges in lesion delineation. MRI emerged as the beacon of precision, especially within dense tissue, though it occasionally grappled with false positives. While each modality possesses its unique strengths and challenges, the holistic synthesis underscores the multifaceted nature of dense breast tissue imaging, warranting a wise blend of modalities for optimal patient outcomes.

Discussion

The ever-evolving landscape of radiological techniques has consistently championed the accurate detection and subsequent management of various pathologies. Breast carcinoma, given its prominence in oncological diagnoses, particularly within dense breast tissue, has been at the forefront of such advancements. A systematic review was undertaken, examining a plethora of studies, and the ensuing results offer illuminative insights. This discussion delves into these findings, critically appraising their implications in the realm of diagnostic radiology.

Comparative Analysis of Imaging Modalities

Mammography has, over the years, demonstrated varying degrees of success in lesion detection within dense breast tissue. This study reviewed seminal works, with Berg et al. [16] showing a specificity of 84% and Autier et al. [20] presenting a sensitivity rate of 77%. While these figures are commendable, it’s paramount to view them in the larger clinical milieu. The average accuracy rates, aggregated from the chosen studies, indeed echo the efficacy of mammography. However, it's crucial to consider the complexities of the age demographics it serves best. Lee et al. [11]'s study focused on an age demographic of 35-82, yielding a sensitivity of 93.1%. Comparatively, Berg et al. [16] which reported a 84% accuracy rate catered to the 25-91 age bracket. These slight discrepancies underscore the potential variability of mammographic results across age cohorts. Another concern, occasionally overshadowed by raw accuracy metrics, is the rate of false negatives. Gilbert and Pinker-Domenig [22] indicated a sensitivity rate of 70%, implicitly highlighting that a notable fraction of carcinomas might evade detection. For a consultant radiologist, this statistic isn't merely a number; it translates into potential missed diagnoses, necessitating the augmentation of mammographic results with other modalities or clinical assessments.

Ultrasound: In the armamentarium of breast imaging, ultrasound has carved a niche for its ability to differentiate lesions, especially in scenarios where mammography encounters challenges. Berg et al. [16] accentuated ultrasound's prowess, presenting an impressive differentiation accuracy of 84%. Yet, like any modality, ultrasound isn't without its challenges. Tagliafico et al. [15] highlighted the modality's occasional struggle in differentiating benign from malignant lesions, leading to potential false positives. Such outcomes mandate a nuanced approach in lesion interpretation, weighing the risks of overtreatment against the peril of missed malignant lesions.

MRI: Often lauded for its precision, especially in contexts demanding intricate tissue differentiation. Chen et al. [14], echoed this sentiment. Such outcomes, particularly in dense breast tissue, signify MRI's potential as a diagnostic stalwart. However, MRI’s excellence in lesion characterization is occasionally offset by its false-positive rates. Tilanus-Linthorst et al. [18] and Menezes et al. [21] commented on this propensity, despite MRI's substantial accuracy in carcinoma differentiation. These false positives, though statistically minor, have profound clinical ramifications, ranging from unnecessary biopsies to the psychological distress they might induce in patients.

In summation, as diagnostic radiologists, we're always poised at the connection of technological advancements and their clinical implications. The presented studies, with their multifaceted outcomes, accentuate the nuanced role each modality plays in breast carcinoma detection within dense breast tissue. Harnessing their individual strengths, understanding their limitations, and integrating them will indubitably lead us towards optimal patient outcomes.

Clinical Implications

Influence on clinical decisions: The clinical arena of radiology, despite being jammed with technological marvels, is always subjected to the acid test of real-world diagnoses. This systematic review provided insights that signal certain shifts in clinical decision-making, especially when contending with dense breast tissue. For instance, mammography, despite its ubiquity, was evidenced to occasionally falter in high-density scenarios [12]. This perhaps underlines a more judicious deployment of mammography, ensuring its integration with patient's age, breast density, and potential risk factors.

Dual or tri-modality imaging: Drawing from the review’s revelations, there emerges a major dialogue around dual or tri-modality imaging. While the robustness of MRI in lesion differentiation was emphasized [12], its heightened false-positive rates cannot be overlooked [22]. Ultrasound, with its tremendous differentiation ability, could perhaps serve as a synergistic companion, ensuring that MRI's potential false positives are accurately evaluated.

Consequences of false outcomes: Radiological diagnoses are not merely pixelated representations; they wield implications that falls into therapeutic decisions, patient well-being, and even the psychological landscape. The false negatives could accidentally cause missed carcinomas, a scenario riddled with significant morbidity. On the other spectrum, the false positives, particularly highlighted in the context of MRI [21], translate into unwarranted biopsies and the psychological burden they impose upon patients. Such findings underscore the imperative for robust interpretative skills, backed by a sensible deployment of imaging modalities.

Study Methodology Considerations

Study designs and implications: The present systematic review incorporated a melange of RCTs and observational studies, each wielding its methodological character. While RCTs are celebrated for their capacity to attenuate confounding biases [20], observational studies often offer the breadth of real-world data [16]. It's crucial, thus, for radiologists to appreciate these nuances, understanding that the synthesized outcomes are an amalgam of tightly controlled environments and broader clinical landscapes.

Population characteristics and generalizability: The universality of radiological practices is often at odds with the geographical and demographic idiosyncrasies that studies encapsulate. Sumkin et al. [17], focusing on American cohorts, might offer results by regional clinical practices or genetic predispositions. This tapestry of populations, while enriching the review, mandates a nuanced interpretation, ensuring that findings are appropriately contextualized within the purview of one's clinical domain.

Sample sizes, statistical power and conclusions: The bedrock of any credible research is invariably the statistical power, which is intricately linked to sample sizes. Studies with robust cohorts, like that of [14], offer a greater confidence in the results’ generalizability. However, it's paramount for consultant radiologists to discern between studies with robust power and those that might offer tentative insights, ensuring that clinical decisions are predicated upon robust evidence.

Bias Analysis Deep Dive

Implications of detected biases: In the realm of research, biases aren't mere methodological footnotes; they sculpt the findings and their subsequent interpretations. This review, while being comprehensive, unearthed certain biases in individual studies. The implications of such biases, be it selection or confirmation biases, have the potential to skew results, and a nuanced appreciation of these is essential for a holistic understanding [13].

Publication and regional biases’ influence: Beyond the scope of individual studies, there lurk the spectres of publication and regional biases. Often, positive outcomes find their way to prominent journals, while negative or neutral results languish in obscurity. Regional biases, fueled by geographical preferences in research focus, further complicate the landscape. Such biases could inadvertently sculpt the perceptions of the radiology community, emphasizing the imperative for a judicious consumption of literature.

Unique insights and contrasts: Yet, amidst these harmonies, there emerge distinct notes, rendering this review both a validation and a challenge to the status quo. The nuanced insights into the potential of dual or tri-modality imaging, predicated upon the intricate interplay of the three modalities, offer a fresh perspective, nudging the radiology community towards an integrated diagnostic paradigm.

Limitations

Acknowledgment of research constraints: No research, regardless of its methodological robustness or the breadth of its review, is entirely devoid of limitations. This systematic review, despite its comprehensive assessment of mammography, ultrasound, and MRI, bears certain inherent constraints. Recognizing these limitations not only amplifies the transparency of the research but underscores the nuances within which the findings should be contextualized [23].

Methodological diversity: One of the more pronounced limitations stems from the heterogeneous methodologies embraced by the included studies. While some research ventures leaned heavily into rigorous RCTs, others navigated the landscape through observational explorations. This methodological diversity, while enriching the review with varied perspectives, introduces an element of variability, with potential implications on the synthesized outcomes [24]. Such a melange mandates an interpretative prudence, ensuring that the aggregate outcomes are judiciously consumed in clinical realms.

Geographical heterogeneity: The global theater of medical research is characterized by a rich tapestry of clinical practices, genetic predispositions, and healthcare infrastructure. The studies included in this review span diverse geographical domains, each bearing its unique clinical imprints. While such diversity augments the comprehensiveness, it also introduces nuances that might temper global applicability. For instance, findings from a European cohort [19] might be nuanced differently when juxtaposed against an Asian demographic, underscoring the imperative for geographically contextualized interpretations.

Recommendations for Future Research

Geographical inclusivity: The horizon of breast imaging research beckons a more geographically inclusive paradigm. Given the demographic nuances in breast density and carcinoma presentations, future endeavors could benefit immensely from studies that transcend continental boundaries, ensuring that findings are not just universal but are also resonant with localized clinical challenges [25].

Exploring novel imaging frontiers: The trinity of mammography, ultrasound, and MRI, while formidable, should not preclude explorations into newer imaging horizons. As technology gallops forward, the potential incorporation of artificial intelligence in lesion differentiation or even the foray into elastography and thermography could further amplify diagnostic precision [26]. Moreover, modifications to extant modalities, ensuring they are attuned to the challenges of dense breast tissue, might be worthwhile avenues for future investigations.

Emphasis on multi-centric trials: Amplifying the clarion call for more robust research designs, the radiology community could be significantly bolstered by larger multi-centric trials. Such endeavors, by spanning varied healthcare ecosystems and patient demographics, not only elevate the generalizability of findings but also offer insights tempered by diverse clinical practices [19].

Final Synthesis

Revisiting pivotal revelations: As this systematic review navigates its closure, revisiting the pivotal findings elucidates the intricate choreography of mammography, ultrasound, and MRI in the landscape of dense breast tissue carcinoma detection. Each modality, with its unique strengths and potential pitfalls, offers a palette of diagnostic insights that are both clinically relevant and imperative for patient management [27].

Tailored radiological approaches: Emerging from the synthesized insights, there reverberates a call for a more tailored approach in radiological practices. By adeptly integrating the strengths of each modality, and being astutely aware of their limitations, the realm of diagnostic radiology can truly transcend its current horizons, ensuring that patient care is not just optimal but is also predicated upon the bedrock of evidence-based practices [28].

## Conclusions

In the intricate realm of diagnostic radiology, the endeavor to identify the most efficacious method for detecting breast carcinoma within dense breast tissue is of utmost importance. This systematic review meticulously examined the merits and limitations of mammography, ultrasound, and MRI within this challenging clinical scenario. The analysis highlighted the nuanced roles of these modalities: mammography, despite its traditional importance, faces limitations in dense breast tissue; ultrasound, with its advantage of non-ionizing radiation and real-time imaging, showed superiority in differentiation within certain demographic cohorts; MRI, known for its precision, carries the drawback of elevated false positive rates, mandating cautious interpretation.

A single imaging modality's dominance is not established; rather, the findings support a strategic combination of modalities, tailored to each patient's unique profile, breast density, and specific clinical context. This holistic approach aims to enhance detection rates while reducing diagnostic ambiguities, emphasizing the primary goal of patient well-being. Thus, this review significantly contributes to the field of breast imaging research, advocating for a patient-centered imaging strategy in the continuously evolving landscape of diagnostic radiology. The ultimate beneficiary is the patient, who gains from the combined expertise and technological advancements in the field.
